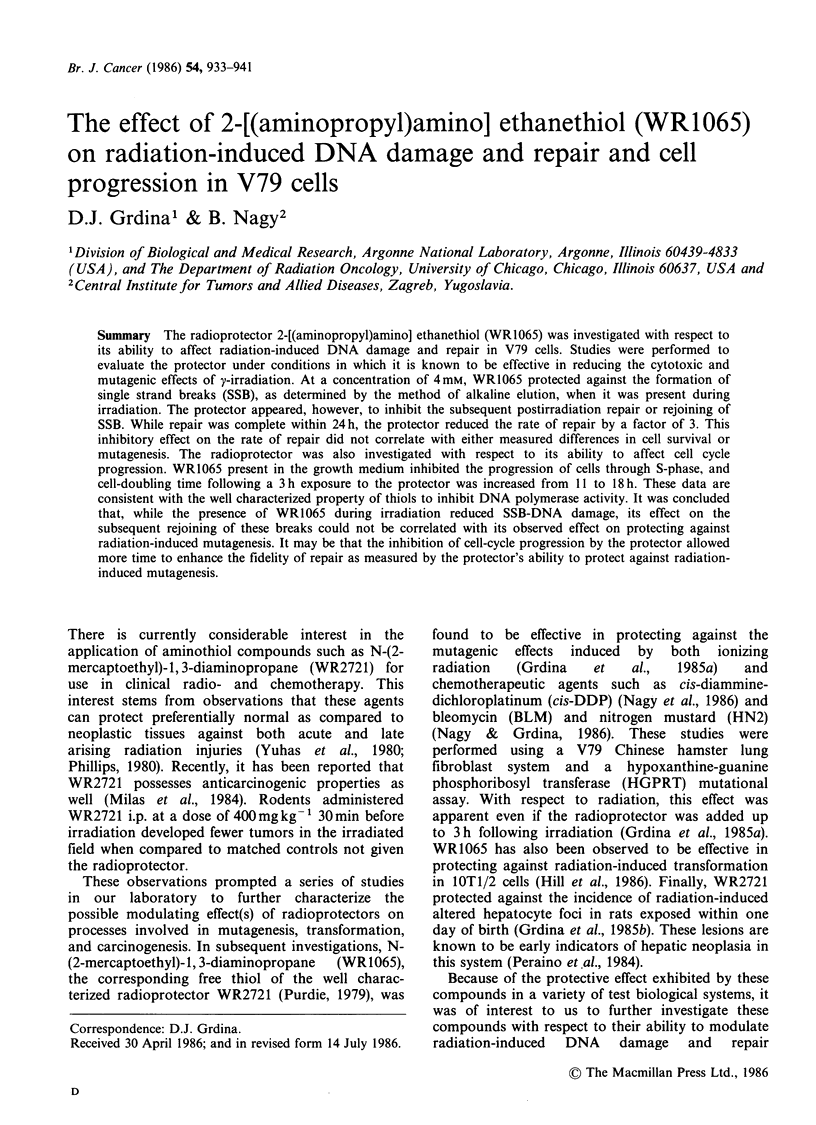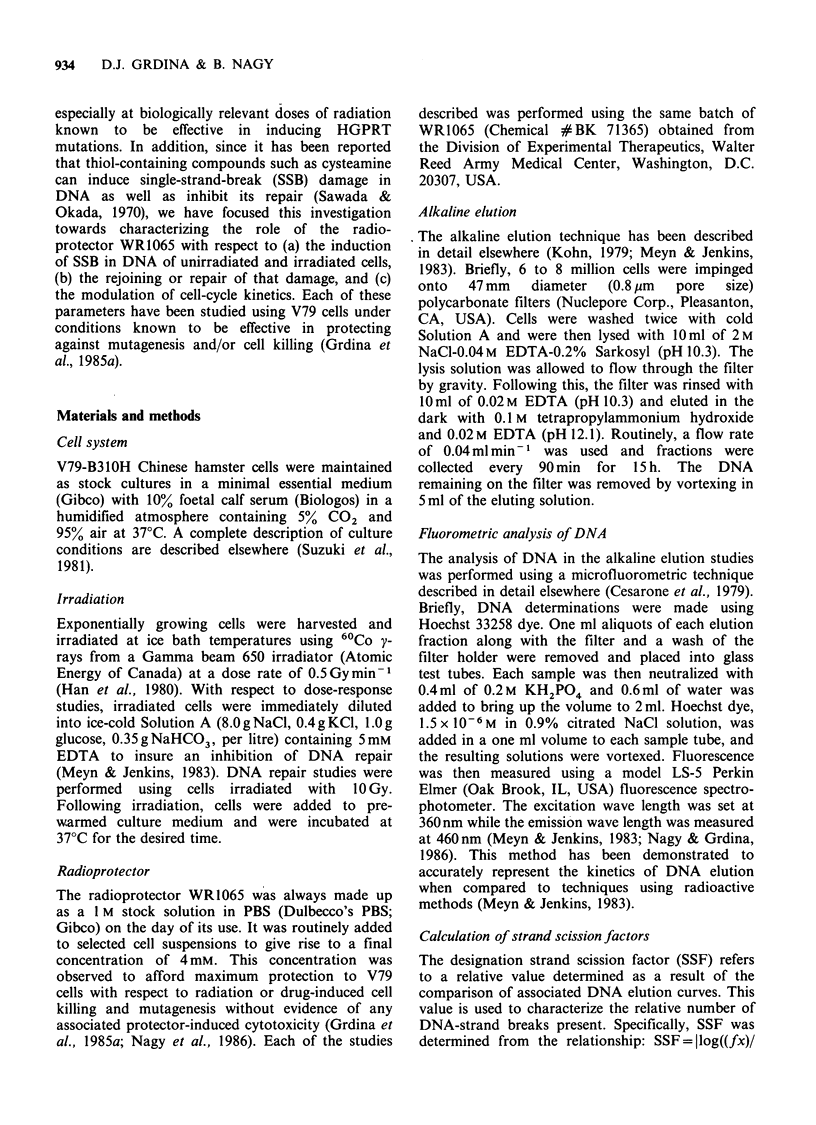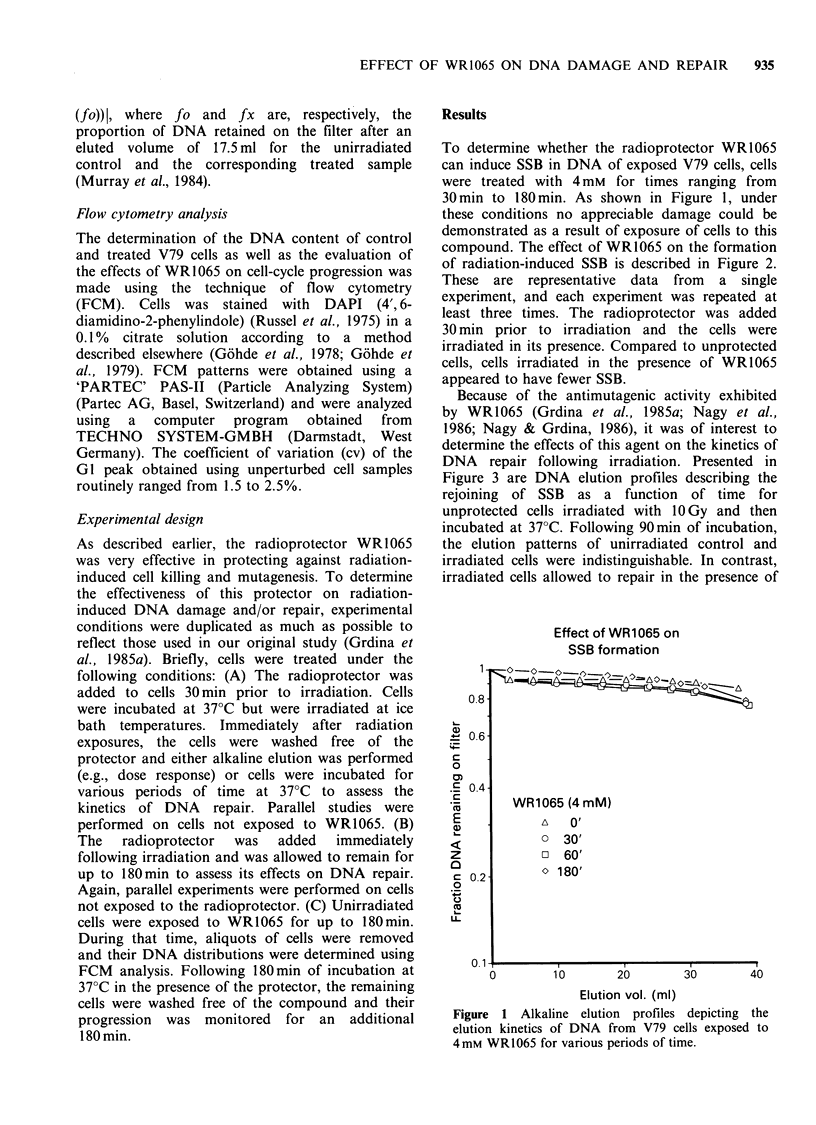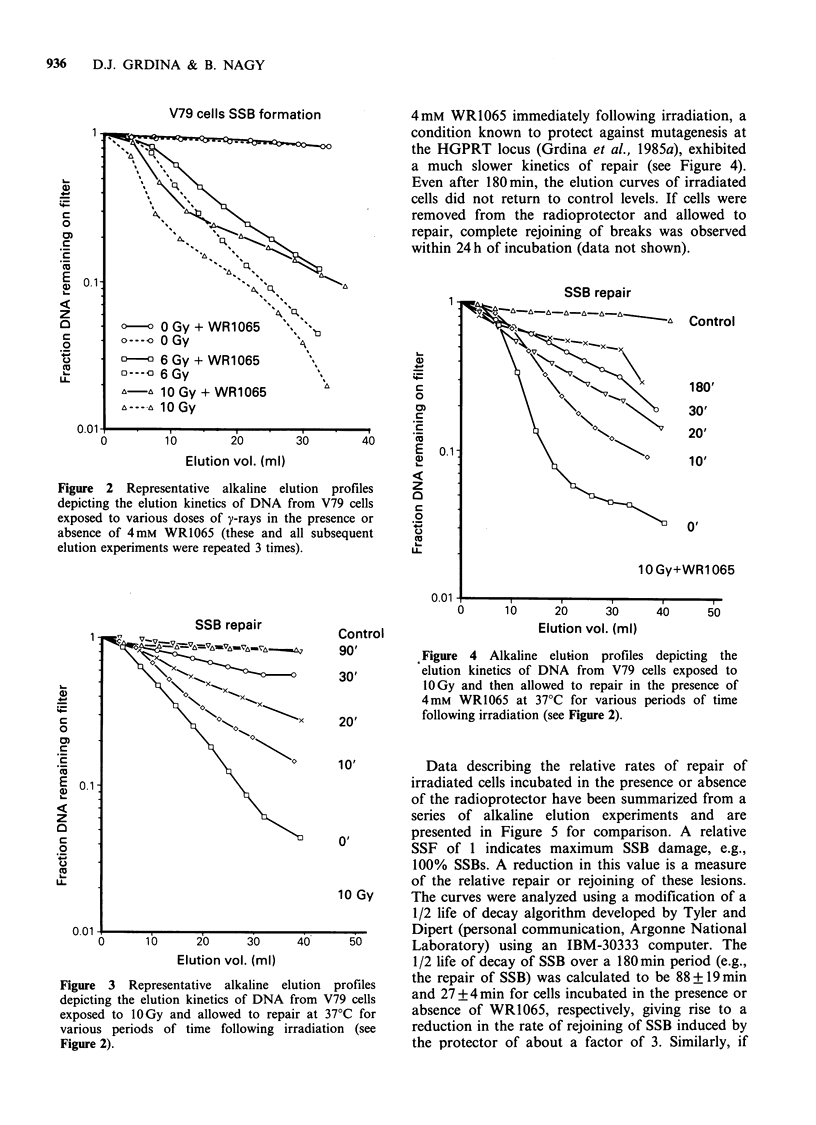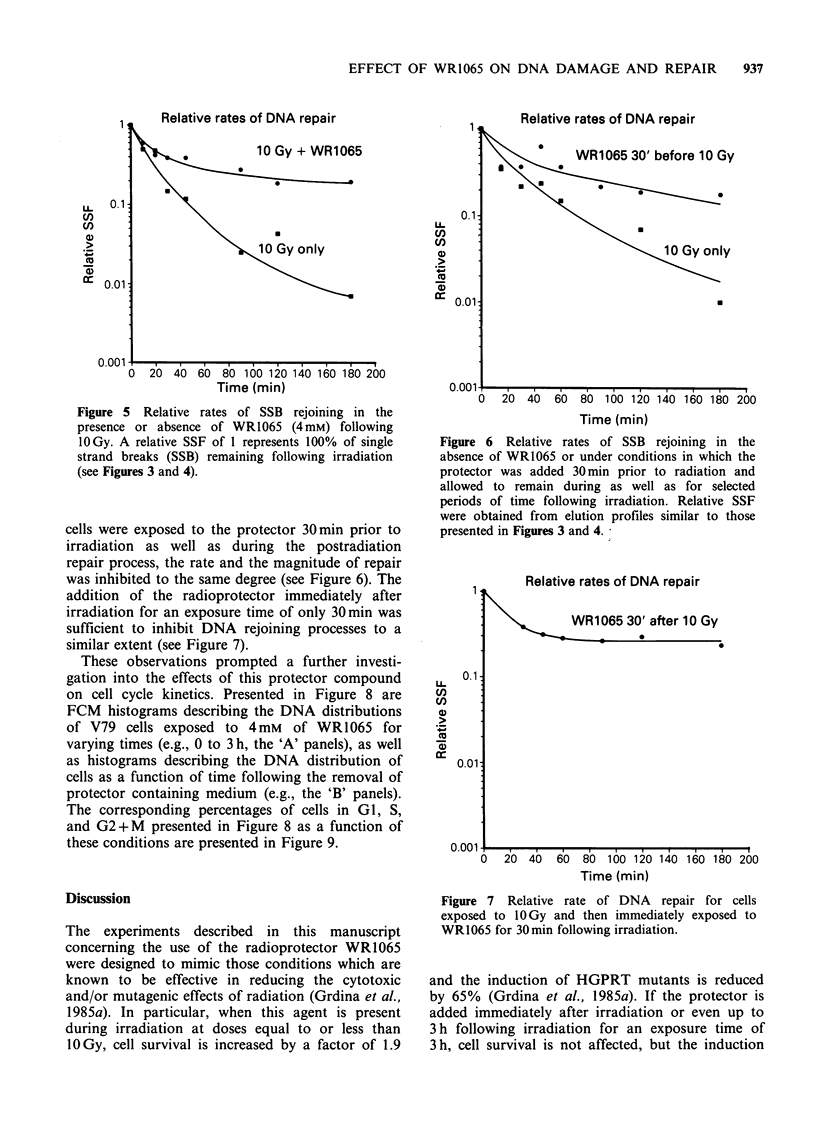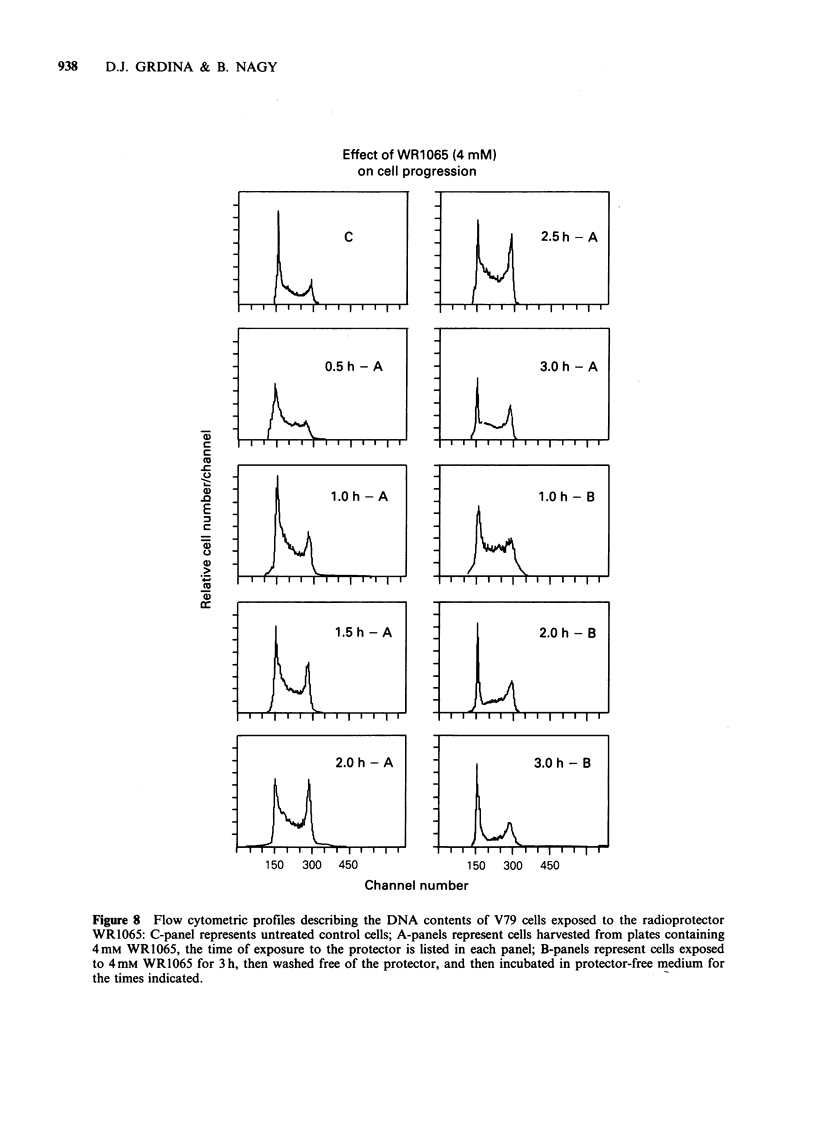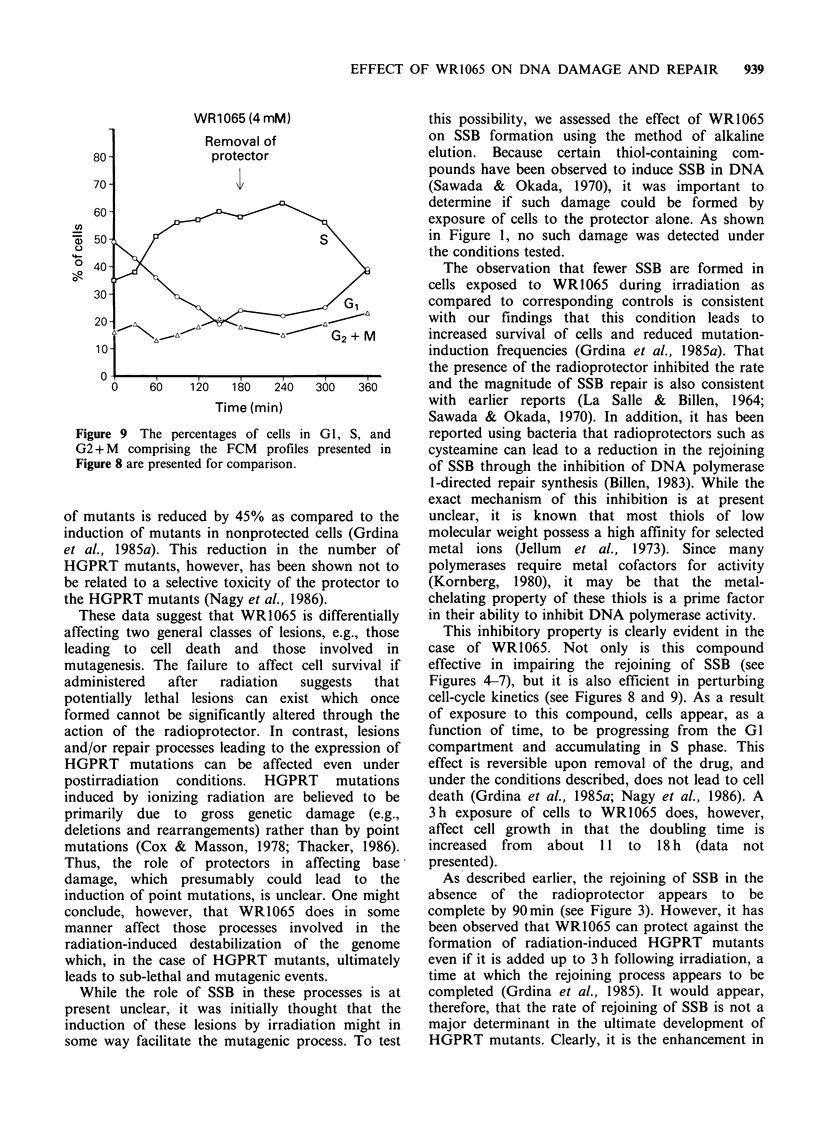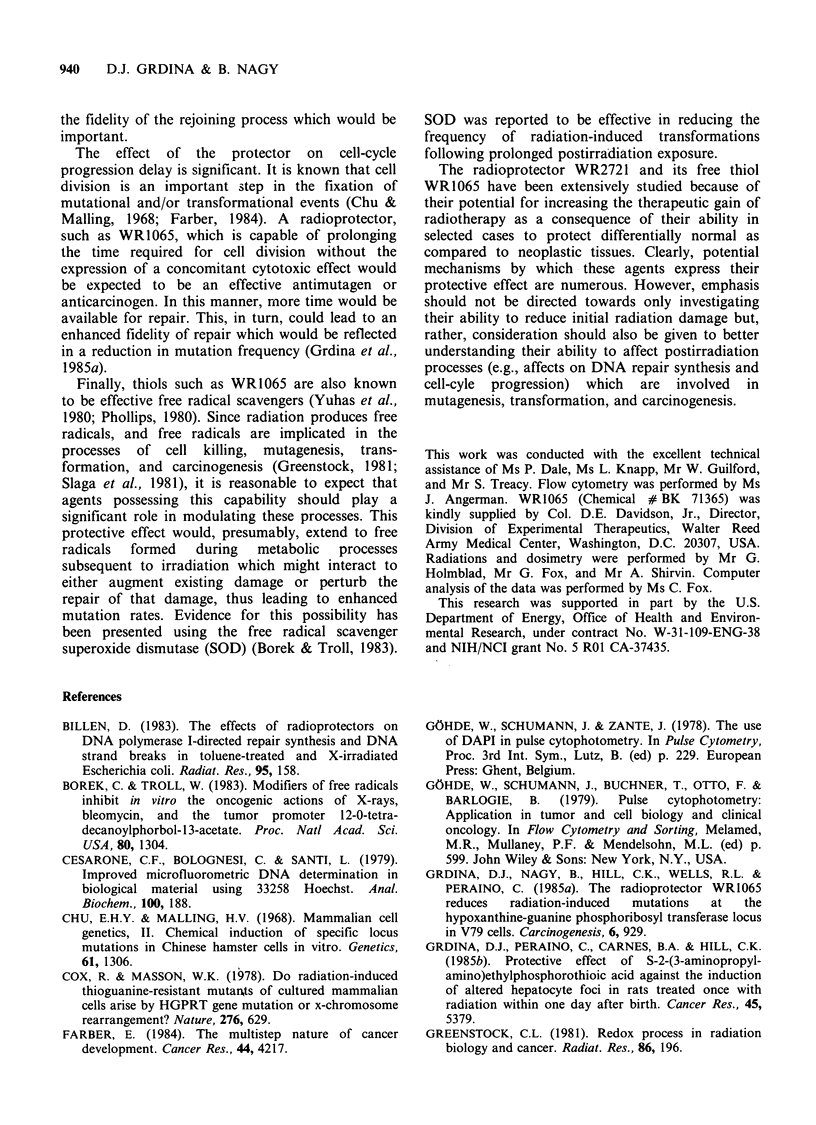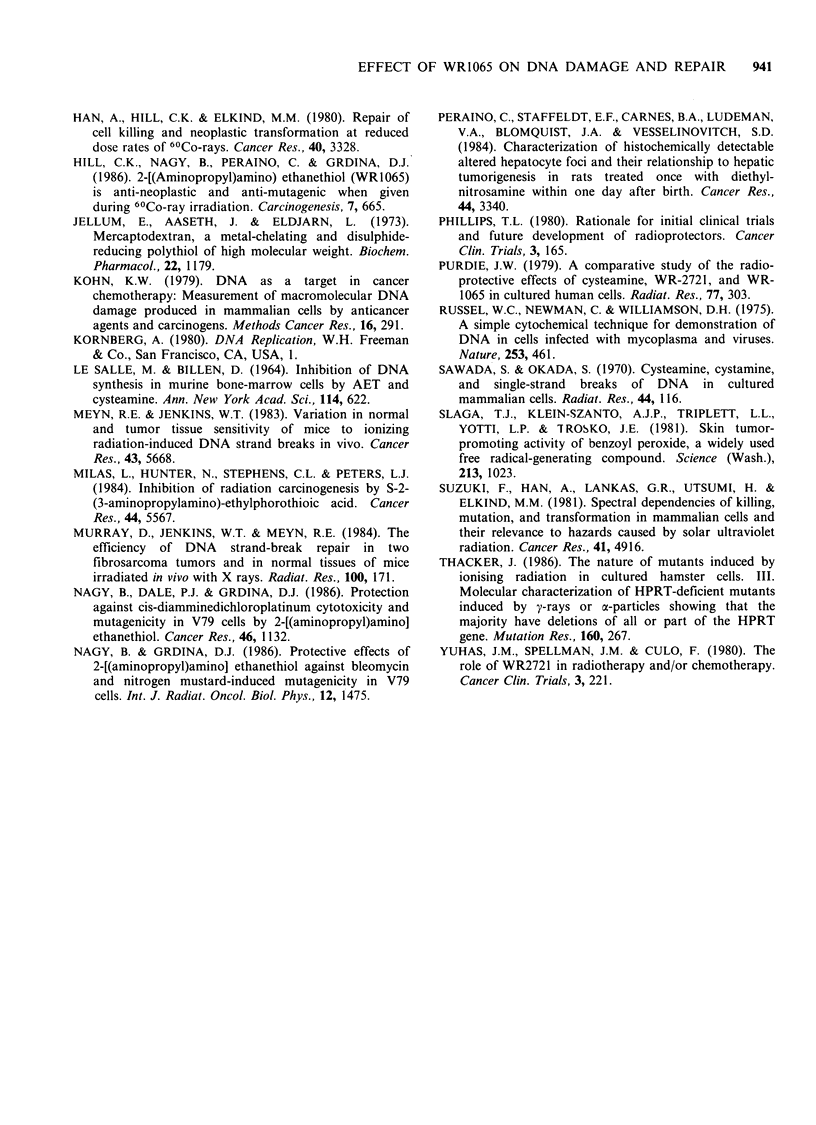# The effect of 2-[(aminopropyl)amino] ethanethiol (WR1065) on radiation-induced DNA damage and repair and cell progression in V79 cells.

**DOI:** 10.1038/bjc.1986.264

**Published:** 1986-12

**Authors:** D. J. Grdina, B. Nagy

## Abstract

The radioprotector 2-[(aminopropyl)amino] ethanethiol (WR1065) was investigated with respect to its ability to affect radiation-induced DNA damage and repair in V79 cells. Studies were performed to evaluate the protector under conditions in which it is known to be effective in reducing the cytotoxic and mutagenic effects of gamma-irradiation. At a concentration of 4 mM, WR1065 protected against the formation of single strand breaks (SSB), as determined by the method of alkaline elution, when it was present during irradiation. The protector appeared, however, to inhibit the subsequent postirradiation repair or rejoining of SSB. While repair was complete within 24 h, the protector reduced the rate of repair by a factor of 3. This inhibitory effect on the rate of repair did not correlate with either measured differences in cell survival or mutagenesis. The radioprotector was also investigated with respect to its ability to affect cell cycle progression. WR1065 present in the growth medium inhibited the progression of cells through S-phase, and cell-doubling time following a 3 h exposure to the protector was increased from 11 to 18 h. These data are consistent with the well characterized property of thiols to inhibit DNA polymerase activity. It was concluded that, while the presence of WR1065 during irradiation reduced SSB-DNA damage, its effect on the subsequent rejoining of these breaks could not be correlated with its observed effect on protecting against radiation-induced mutagenesis. It may be that the inhibition of cell-cycle progression by the protector allowed more time to enhance the fidelity of repair as measured by the protector's ability to protect against radiation-induced mutagenesis.


*Br. J. Cancer* (1986) **54**, 933–941

### The effect of 2-[(aminopropyl)amino] ethanethiol (WR1065) on radiation-induced DNA damage and repair and cell progression in V79 cells

D.J. Grdina[1] & B. Nagy[2]

[1]*Division of Biological and Medical Research, Argonne National Laboratory, Argonne, Illinois 60439-4833 (USA), and The Department of Radiation Oncology, University of Chicago, Chicago, Illinois 60637, USA and* [2]*Central Institute for Tumors and Allied Diseases, Zagreb, Yugoslavia.*

**Summary** The radioprotector 2-[(aminopropyl)amino] ethanethiol (WR1065) was investigated with respect to its ability to affect radiation-induced DNA damage and repair in V79 cells. Studies were performed to evaluate the protector under conditions in which it is known to be effective in reducing the cytotoxic and mutagenic effects of $\gamma$-irradiation. At a concentration of 4 mM, WR1065 protected against the formation of single strand breaks (SSB), as determined by the method of alkaline elution, when it was present during irradiation. The protector appeared, however, to inhibit the subsequent postirradiation repair or rejoining of SSB. While repair was complete within 24 h, the protector reduced the rate of repair by a factor of 3. This inhibitory effect on the rate of repair did not correlate with either measured differences in cell survival or mutagenesis. The radioprotector was also investigated with respect to its ability to affect cell cycle progression. WR1065 present in the growth medium inhibited the progression of cells through S-phase, and cell-doubling time following a 3 h exposure to the protector was increased from 11 to 18 h. These data are consistent with the well characterized property of thiols to inhibit DNA polymerase activity. It was concluded that, while the presence of WR1065 during irradiation reduced SSB-DNA damage, its effect on the subsequent rejoining of these breaks could not be correlated with its observed effect on protecting against radiation-induced mutagenesis. It may be that the inhibition of cell-cycle progression by the protector allowed more time to enhance the fidelity of repair as measured by the protector's ability to protect against radiation-induced mutagenesis.

There is currently considerable interest in the application of aminothiol compounds such as N-(2-mercaptoethyl)-1, 3-diaminopropane (WR2721) for use in clinical radio- and chemotherapy. This interest stems from observations that these agents can protect preferentially normal as compared to neoplastic tissues against both acute and late arising radiation injuries (Yuhas *et al.*, 1980; Phillips, 1980). Recently, it has been reported that WR2721 possesses anticarcinogenic properties as well (Milas *et al.*, 1984). Rodents administered WR2721 i.p. at a dose of 400 mg kg$^{-1}$ 30 min before irradiation developed fewer tumors in the irradiated field when compared to matched controls not given the radioprotector.

These observations prompted a series of studies in our laboratory to further characterize the possible modulating effect(s) of radioprotectors on processes involved in mutagenesis, transformation, and carcinogenesis. In subsequent investigations, N-(2-mercaptoethyl)-1, 3-diaminopropane (WR1065), the corresponding free thiol of the well characterized radioprotector WR2721 (Purdie, 1979), was found to be effective in protecting against the mutagenic effects induced by both ionizing radiation (Grdina *et al.*, 1985*a*) and chemotherapeutic agents such as *cis*-diammine-dichloroplatinum (*cis*-DDP) (Nagy *et al.*, 1986) and bleomycin (BLM) and nitrogen mustard (HN2) (Nagy & Grdina, 1986). These studies were performed using a V79 Chinese hamster lung fibroblast system and a hypoxanthine-guanine phosphoribosyl transferase (HGPRT) mutational assay. With respect to radiation, this effect was apparent even if the radioprotector was added up to 3 h following irradiation (Grdina *et al.*, 1985*a*). WR1065 has also been observed to be effective in protecting against radiation-induced transformation in 10T1/2 cells (Hill *et al.*, 1986). Finally, WR2721 protected against the incidence of radiation-induced altered hepatocyte foci in rats exposed within one day of birth (Grdina *et al.*, 1985*b*). These lesions are known to be early indicators of hepatic neoplasia in this system (Peraino *et al.*, 1984).

Because of the protective effect exhibited by these compounds in a variety of test biological systems, it was of interest to us to further investigate these compounds with respect to their ability to modulate radiation-induced DNA damage and repair

Correspondence: D.J. Grdina.
Received 30 April 1986; and in revised form 14 July 1986.

© The Macmillan Press Ltd., 1986

especially at biologically relevant doses of radiation known to be effective in inducing HGPRT mutations. In addition, since it has been reported that thiol-containing compounds such as cysteamine can induce single-strand-break (SSB) damage in DNA as well as inhibit its repair (Sawada & Okada, 1970), we have focused this investigation towards characterizing the role of the radioprotector WR1065 with respect to (a) the induction of SSB in DNA of unirradiated and irradiated cells, (b) the rejoining or repair of that damage, and (c) the modulation of cell-cycle kinetics. Each of these parameters have been studied using V79 cells under conditions known to be effective in protecting against mutagenesis and/or cell killing (Grdina *et al.*, 1985*a*).

### Materials and methods

### *Cell system*

V79-B310H Chinese hamster cells were maintained as stock cultures in a minimal essential medium (Gibco) with 10% foetal calf serum (Biologos) in a humidified atmosphere containing 5% $CO_2$ and 95% air at 37°C. A complete description of culture conditions are described elsewhere (Suzuki *et al.*, 1981).

### *Irradiation*

Exponentially growing cells were harvested and irradiated at ice bath temperatures using $^{60}$Co $\gamma$-rays from a Gamma beam 650 irradiator (Atomic Energy of Canada) at a dose rate of 0.5 Gy min$^{-1}$ (Han *et al.*, 1980). With respect to dose-response studies, irradiated cells were immediately diluted into ice-cold Solution A (8.0 g NaCl, 0.4 g KCl, 1.0 g glucose, 0.35 g $NaHCO_3$, per litre) containing 5 mM EDTA to insure an inhibition of DNA repair (Meyn & Jenkins, 1983). DNA repair studies were performed using cells irradiated with 10 Gy. Following irradiation, cells were added to pre-warmed culture medium and were incubated at 37°C for the desired time.

### *Radioprotector*

The radioprotector WR1065 was always made up as a 1 M stock solution in PBS (Dulbecco's PBS; Gibco) on the day of its use. It was routinely added to selected cell suspensions to give rise to a final concentration of 4 mM. This concentration was observed to afford maximum protection to V79 cells with respect to radiation or drug-induced cell killing and mutagenesis without evidence of any associated protector-induced cytotoxicity (Grdina *et al.*, 1985*a*; Nagy *et al.*, 1986). Each of the studies described was performed using the same batch of WR1065 (Chemical #BK 71365) obtained from the Division of Experimental Therapeutics, Walter Reed Army Medical Center, Washington, D.C. 20307, USA.

### *Alkaline elution*

The alkaline elution technique has been described in detail elsewhere (Kohn, 1979; Meyn & Jenkins, 1983). Briefly, 6 to 8 million cells were impinged onto 47 mm diameter (0.8 $\mu$m pore size) polycarbonate filters (Nuclepore Corp., Pleasanton, CA, USA). Cells were washed twice with cold Solution A and were then lysed with 10 ml of 2 M NaCl-0.04 M EDTA-0.2% Sarkosyl (pH 10.3). The lysis solution was allowed to flow through the filter by gravity. Following this, the filter was rinsed with 10 ml of 0.02 M EDTA (pH 10.3) and eluted in the dark with 0.1 M tetrapropylammonium hydroxide and 0.02 M EDTA (pH 12.1). Routinely, a flow rate of 0.04 ml min$^{-1}$ was used and fractions were collected every 90 min for 15 h. The DNA remaining on the filter was removed by vortexing in 5 ml of the eluting solution.

### *Fluorometric analysis of DNA*

The analysis of DNA in the alkaline elution studies was performed using a microfluorometric technique described in detail elsewhere (Cesarone *et al.*, 1979). Briefly, DNA determinations were made using Hoechst 33258 dye. One ml aliquots of each elution fraction along with the filter and a wash of the filter holder were removed and placed into glass test tubes. Each sample was then neutralized with 0.4 ml of 0.2 M $KH_2PO_4$ and 0.6 ml of water was added to bring up the volume to 2 ml. Hoechst dye, $1.5 \times 10^{-6}$ M in 0.9% citrated NaCl solution, was added in a one ml volume to each sample tube, and the resulting solutions were vortexed. Fluorescence was then measured using a model LS-5 Perkin Elmer (Oak Brook, IL, USA) fluorescence spectrophotometer. The excitation wave length was set at 360 nm while the emission wave length was measured at 460 nm (Meyn & Jenkins, 1983; Nagy & Grdina, 1986). This method has been demonstrated to accurately represent the kinetics of DNA elution when compared to techniques using radioactive methods (Meyn & Jenkins, 1983).

### *Calculation of strand scission factors*

The designation strand scission factor (SSF) refers to a relative value determined as a result of the comparison of associated DNA elution curves. This value is used to characterize the relative number of DNA-strand breaks present. Specifically, SSF was determined from the relationship: SSF = |log((*fx*)/

$(fo))|$, where $fo$ and $fx$ are, respectively, the proportion of DNA retained on the filter after an eluted volume of 17.5 ml for the unirradiated control and the corresponding treated sample (Murray *et al.*, 1984).

*Flow cytometry analysis*

The determination of the DNA content of control and treated V79 cells as well as the evaluation of the effects of WR1065 on cell-cycle progression was made using the technique of flow cytometry (FCM). Cells was stained with DAPI (4′,6-diamidino-2-phenylindole) (Russel *et al.*, 1975) in a 0.1% citrate solution according to a method described elsewhere (Göhde *et al.*, 1978; Göhde *et al.*, 1979). FCM patterns were obtained using a 'PARTEC' PAS-II (Particle Analyzing System) (Partec AG, Basel, Switzerland) and were analyzed using a computer program obtained from TECHNO SYSTEM-GMBH (Darmstadt, West Germany). The coefficient of variation (cv) of the G1 peak obtained using unperturbed cell samples routinely ranged from 1.5 to 2.5%.

*Experimental design*

As described earlier, the radioprotector WR1065 was very effective in protecting against radiation-induced cell killing and mutagenesis. To determine the effectiveness of this protector on radiation-induced DNA damage and/or repair, experimental conditions were duplicated as much as possible to reflect those used in our original study (Grdina *et al.*, 1985*a*). Briefly, cells were treated under the following conditions: (A) The radioprotector was added to cells 30 min prior to irradiation. Cells were incubated at 37°C but were irradiated at ice bath temperatures. Immediately after radiation exposures, the cells were washed free of the protector and either alkaline elution was performed (e.g., dose response) or cells were incubated for various periods of time at 37°C to assess the kinetics of DNA repair. Parallel studies were performed on cells not exposed to WR1065. (B) The radioprotector was added immediately following irradiation and was allowed to remain for up to 180 min to assess its effects on DNA repair. Again, parallel experiments were performed on cells not exposed to the radioprotector. (C) Unirradiated cells were exposed to WR1065 for up to 180 min. During that time, aliquots of cells were removed and their DNA distributions were determined using FCM analysis. Following 180 min of incubation at 37°C in the presence of the protector, the remaining cells were washed free of the compound and their progression was monitored for an additional 180 min.

**Results**

To determine whether the radioprotector WR1065 can induce SSB in DNA of exposed V79 cells, cells were treated with 4 mM for times ranging from 30 min to 180 min. As shown in Figure 1, under these conditions no appreciable damage could be demonstrated as a result of exposure of cells to this compound. The effect of WR1065 on the formation of radiation-induced SSB is described in Figure 2. These are representative data from a single experiment, and each experiment was repeated at least three times. The radioprotector was added 30 min prior to irradiation and the cells were irradiated in its presence. Compared to unprotected cells, cells irradiated in the presence of WR1065 appeared to have fewer SSB.

Because of the antimutagenic activity exhibited by WR1065 (Grdina *et al.*, 1985*a*; Nagy *et al.*, 1986; Nagy & Grdina, 1986), it was of interest to determine the effects of this agent on the kinetics of DNA repair following irradiation. Presented in Figure 3 are DNA elution profiles describing the rejoining of SSB as a function of time for unprotected cells irradiated with 10 Gy and then incubated at 37°C. Following 90 min of incubation, the elution patterns of unirradiated control and irradiated cells were indistinguishable. In contrast, irradiated cells allowed to repair in the presence of

**Figure 1** Alkaline elution profiles depicting the elution kinetics of DNA from V79 cells exposed to 4 mM WR1065 for various periods of time.

**Figure 2** Representative alkaline elution profiles depicting the elution kinetics of DNA from V79 cells exposed to various doses of $\gamma$-rays in the presence or absence of 4mM WR1065 (these and all subsequent elution experiments were repeated 3 times).

**Figure 3** Representative alkaline elution profiles depicting the elution kinetics of DNA from V79 cells exposed to 10 Gy and allowed to repair at 37°C for various periods of time following irradiation (see **Figure 2**).

4mM WR1065 immediately following irradiation, a condition known to protect against mutagenesis at the HGPRT locus (Grdina *et al.*, 1985*a*), exhibited a much slower kinetics of repair (see Figure 4). Even after 180 min, the elution curves of irradiated cells did not return to control levels. If cells were removed from the radioprotector and allowed to repair, complete rejoining of breaks was observed within 24 h of incubation (data not shown).

**Figure 4** Alkaline elution profiles depicting the elution kinetics of DNA from V79 cells exposed to 10 Gy and then allowed to repair in the presence of 4mM WR1065 at 37°C for various periods of time following irradiation (see **Figure 2**).

Data describing the relative rates of repair of irradiated cells incubated in the presence or absence of the radioprotector have been summarized from a series of alkaline elution experiments and are presented in Figure 5 for comparison. A relative SSF of 1 indicates maximum SSB damage, e.g., 100% SSBs. A reduction in this value is a measure of the relative repair or rejoining of these lesions. The curves were analyzed using a modification of a 1/2 life of decay algorithm developed by Tyler and Dipert (personal communication, Argonne National Laboratory) using an IBM-30333 computer. The 1/2 life of decay of SSB over a 180 min period (e.g., the repair of SSB) was calculated to be $88 \pm 19$ min and $27 \pm 4$ min for cells incubated in the presence or absence of WR1065, respectively, giving rise to a reduction in the rate of rejoining of SSB induced by the protector of about a factor of 3. Similarly, if

**Figure 5** Relative rates of SSB rejoining in the presence or absence of WR1065 (4 mM) following 10 Gy. A relative SSF of 1 represents 100% of single strand breaks (SSB) remaining following irradiation (see **Figures** 3 and 4).

**Figure 6** Relative rates of SSB rejoining in the absence of WR1065 or under conditions in which the protector was added 30 min prior to radiation and allowed to remain during as well as for selected periods of time following irradiation. Relative SSF were obtained from elution profiles similar to those presented in **Figures 3** and **4**.

cells were exposed to the protector 30 min prior to irradiation as well as during the postradiation repair process, the rate and the magnitude of repair was inhibited to the same degree (see Figure 6). The addition of the radioprotector immediately after irradiation for an exposure time of only 30 min was sufficient to inhibit DNA rejoining processes to a similar extent (see Figure 7).

These observations prompted a further investigation into the effects of this protector compound on cell cycle kinetics. Presented in Figure 8 are FCM histograms describing the DNA distributions of V79 cells exposed to 4 mM of WR1065 for varying times (e.g., 0 to 3 h, the 'A' panels), as well as histograms describing the DNA distribution of cells as a function of time following the removal of protector containing medium (e.g., the 'B' panels). The corresponding percentages of cells in G1, S, and G2+M presented in Figure 8 as a function of these conditions are presented in Figure 9.

**Figure 7** Relative rate of DNA repair for cells exposed to 10 Gy and then immediately exposed to WR1065 for 30 min following irradiation.

### Discussion

The experiments described in this manuscript concerning the use of the radioprotector WR1065 were designed to mimic those conditions which are known to be effective in reducing the cytotoxic and/or mutagenic effects of radiation (Grdina *et al.*, 1985*a*). In particular, when this agent is present during irradiation at doses equal to or less than 10 Gy, cell survival is increased by a factor of 1.9 and the induction of HGPRT mutants is reduced by 65% (Grdina *et al.*, 1985*a*). If the protector is added immediately after irradiation or even up to 3 h following irradiation for an exposure time of 3 h, cell survival is not affected, but the induction

**Figure 8** Flow cytometric profiles describing the DNA contents of V79 cells exposed to the radioprotector WR1065: C-panel represents untreated control cells; A-panels represent cells harvested from plates containing 4 mM WR1065, the time of exposure to the protector is listed in each panel; B-panels represent cells exposed to 4 mM WR1065 for 3 h, then washed free of the protector, and then incubated in protector-free medium for the times indicated.

**Figure 9** The percentages of cells in G1, S, and G2+M comprising the FCM profiles presented in **Figure 8** are presented for comparison.

of mutants is reduced by 45% as compared to the induction of mutants in nonprotected cells (Grdina *et al.*, 1985*a*). This reduction in the number of HGPRT mutants, however, has been shown not to be related to a selective toxicity of the protector to the HGPRT mutants (Nagy *et al.*, 1986).

These data suggest that WR1065 is differentially affecting two general classes of lesions, e.g., those leading to cell death and those involved in mutagenesis. The failure to affect cell survival if administered after radiation suggests that potentially lethal lesions can exist which once formed cannot be significantly altered through the action of the radioprotector. In contrast, lesions and/or repair processes leading to the expression of HGPRT mutations can be affected even under postirradiation conditions. HGPRT mutations induced by ionizing radiation are believed to be primarily due to gross genetic damage (e.g., deletions and rearrangements) rather than by point mutations (Cox & Masson, 1978; Thacker, 1986). Thus, the role of protectors in affecting base damage, which presumably could lead to the induction of point mutations, is unclear. One might conclude, however, that WR1065 does in some manner affect those processes involved in the radiation-induced destabilization of the genome which, in the case of HGPRT mutants, ultimately leads to sub-lethal and mutagenic events.

While the role of SSB in these processes is at present unclear, it was initially thought that the induction of these lesions by irradiation might in some way facilitate the mutagenic process. To test this possibility, we assessed the effect of WR1065 on SSB formation using the method of alkaline elution. Because certain thiol-containing compounds have been observed to induce SSB in DNA (Sawada & Okada, 1970), it was important to determine if such damage could be formed by exposure of cells to the protector alone. As shown in Figure 1, no such damage was detected under the conditions tested.

The observation that fewer SSB are formed in cells exposed to WR1065 during irradiation as compared to corresponding controls is consistent with our findings that this condition leads to increased survival of cells and reduced mutation-induction frequencies (Grdina *et al.*, 1985*a*). That the presence of the radioprotector inhibited the rate and the magnitude of SSB repair is also consistent with earlier reports (La Salle & Billen, 1964; Sawada & Okada, 1970). In addition, it has been reported using bacteria that radioprotectors such as cysteamine can lead to a reduction in the rejoining of SSB through the inhibition of DNA polymerase 1-directed repair synthesis (Billen, 1983). While the exact mechanism of this inhibition is at present unclear, it is known that most thiols of low molecular weight possess a high affinity for selected metal ions (Jellum *et al.*, 1973). Since many polymerases require metal cofactors for activity (Kornberg, 1980), it may be that the metal-chelating property of these thiols is a prime factor in their ability to inhibit DNA polymerase activity.

This inhibitory property is clearly evident in the case of WR1065. Not only is this compound effective in impairing the rejoining of SSB (see Figures 4–7), but it is also efficient in perturbing cell-cycle kinetics (see Figures 8 and 9). As a result of exposure to this compound, cells appear, as a function of time, to be progressing from the G1 compartment and accumulating in S phase. This effect is reversible upon removal of the drug, and under the conditions described, does not lead to cell death (Grdina *et al.*, 1985*a*; Nagy *et al.*, 1986). A 3 h exposure of cells to WR1065 does, however, affect cell growth in that the doubling time is increased from about 11 to 18 h (data not presented).

As described earlier, the rejoining of SSB in the absence of the radioprotector appears to be complete by 90 min (see Figure 3). However, it has been observed that WR1065 can protect against the formation of radiation-induced HGPRT mutants even if it is added up to 3 h following irradiation, a time at which the rejoining process appears to be completed (Grdina *et al.*, 1985). It would appear, therefore, that the rate of rejoining of SSB is not a major determinant in the ultimate development of HGPRT mutants. Clearly, it is the enhancement in the fidelity of the rejoining process which would be important.

The effect of the protector on cell-cycle progression delay is significant. It is known that cell division is an important step in the fixation of mutational and/or transformational events (Chu & Malling, 1968; Farber, 1984). A radioprotector, such as WR1065, which is capable of prolonging the time required for cell division without the expression of a concomitant cytotoxic effect would be expected to be an effective antimutagen or anticarcinogen. In this manner, more time would be available for repair. This, in turn, could lead to an enhanced fidelity of repair which would be reflected in a reduction in mutation frequency (Grdina *et al.*, 1985*a*).

Finally, thiols such as WR1065 are also known to be effective free radical scavengers (Yuhas *et al.*, 1980; Phollips, 1980). Since radiation produces free radicals, and free radicals are implicated in the processes of cell killing, mutagenesis, transformation, and carcinogenesis (Greenstock, 1981; Slaga *et al.*, 1981), it is reasonable to expect that agents possessing this capability should play a significant role in modulating these processes. This protective effect would, presumably, extend to free radicals formed during metabolic processes subsequent to irradiation which might interact to either augment existing damage or perturb the repair of that damage, thus leading to enhanced mutation rates. Evidence for this possibility has been presented using the free radical scavenger superoxide dismutase (SOD) (Borek & Troll, 1983). SOD was reported to be effective in reducing the frequency of radiation-induced transformations following prolonged postirradiation exposure.

The radioprotector WR2721 and its free thiol WR1065 have been extensively studied because of their potential for increasing the therapeutic gain of radiotherapy as a consequence of their ability in selected cases to protect differentially normal as compared to neoplastic tissues. Clearly, potential mechanisms by which these agents express their protective effect are numerous. However, emphasis should not be directed towards only investigating their ability to reduce initial radiation damage but, rather, consideration should also be given to better understanding their ability to affect postirradiation processes (e.g., affects on DNA repair synthesis and cell-cyle progression) which are involved in mutagenesis, transformation, and carcinogenesis.

This work was conducted with the excellent technical assistance of Ms P. Dale, Ms L. Knapp, Mr W. Guilford, and Mr S. Treacy. Flow cytometry was performed by Ms J. Angerman. WR1065 (Chemical # BK 71365) was kindly supplied by Col. D.E. Davidson, Jr., Director, Division of Experimental Therapeutics, Walter Reed Army Medical Center, Washington, D.C. 20307, USA. Radiations and dosimetry were performed by Mr G. Holmblad, Mr G. Fox, and Mr A. Shirvin. Computer analysis of the data was performed by Ms C. Fox.

This research was supported in part by the U.S. Department of Energy, Office of Health and Environmental Research, under contract No. W-31-109-ENG-38 and NIH/NCI grant No. 5 R01 CA-37435.

### References

BILLEN, D. (1983). The effects of radioprotectors on DNA polymerase I-directed repair synthesis and DNA strand breaks in toluene-treated and X-irradiated Escherichia coli. *Radiat. Res.*, **95**, 158.

BOREK, C. & TROLL, W. (1983). Modifiers of free radicals inhibit *in vitro* the oncogenic actions of X-rays, bleomycin, and the tumor promoter 12-0-tetradecanoylphorbol-13-acetate. *Proc. Natl Acad. Sci. USA*, **80**, 1304.

CESARONE, C.F., BOLOGNESI, C. & SANTI, L. (1979). Improved microfluorometric DNA determination in biological material using 33258 Hoechst. *Anal. Biochem.*, **100**, 188.

CHU, E.H.Y. & MALLING, H.V. (1968). Mammalian cell genetics, II. Chemical induction of specific locus mutations in Chinese hamster cells in vitro. *Genetics*, **61**, 1306.

COX, R. & MASSON, W.K. (1978). Do radiation-induced thioguanine-resistant mutants of cultured mammalian cells arise by HGPRT gene mutation or x-chromosome rearrangement? *Nature*, **276**, 629.

FARBER, E. (1984). The multistep nature of cancer development. *Cancer Res.*, **44**, 4217.

GÖHDE, W., SCHUMANN, J. & ZANTE, J. (1978). The use of DAPI in pulse cytophotometry. In *Pulse Cytometry*, Proc. 3rd Int. Sym., Lutz, B. (ed) p. 229. European Press: Ghent, Belgium.

GÖHDE, W., SCHUMANN, J., BUCHNER, T., OTTO, F. & BARLOGIE, B. (1979). Pulse cytophotometry: Application in tumor and cell biology and clinical oncology. In *Flow Cytometry and Sorting*, Melamed, M.R., Mullaney, P.F. & Mendelsohn, M.L. (ed) p. 599. John Wiley & Sons: New York, N.Y., USA.

GRDINA, D.J., NAGY, B., HILL, C.K., WELLS, R.L. & PERAINO, C. (1985*a*). The radioprotector WR1065 reduces radiation-induced mutations at the hypoxanthine-guanine phosphoribosyl transferase locus in V79 cells. *Carcinogenesis*, **6**, 929.

GRDINA, D.J., PERAINO, C., CARNES, B.A. & HILL, C.K. (1985*b*). Protective effect of S-2-(3-aminopropylamino)ethylphosphorothioic acid against the induction of altered hepatocyte foci in rats treated once with radiation within one day after birth. *Cancer Res.*, **45**, 5379.

GREENSTOCK, C.L. (1981). Redox process in radiation biology and cancer. *Radiat. Res.*, **86**, 196.

HAN, A., HILL, C.K. & ELKIND, M.M. (1980). Repair of cell killing and neoplastic transformation at reduced dose rates of $^{60}$Co-rays. *Cancer Res.*, **40**, 3328.

HILL, C.K., NAGY, B., PERAINO, C. & GRDINA, D.J. (1986). 2-[(Aminopropyl)amino) ethanethiol (WR1065) is anti-neoplastic and anti-mutagenic when given during $^{60}$Co-ray irradiation. *Carcinogenesis*, **7**, 665.

JELLUM, E., AASETH, J. & ELDJARN, L. (1973). Mercaptodextran, a metal-chelating and disulphide-reducing polythiol of high molecular weight. *Biochem. Pharmacol.*, **22**, 1179.

KOHN, K.W. (1979). DNA as a target in cancer chemotherapy: Measurement of macromolecular DNA damage produced in mammalian cells by anticancer agents and carcinogens. *Methods Cancer Res.*, **16**, 291.

KORNBERG, A. (1980). *DNA Replication*, W.H. Freeman & Co., San Francisco, CA, USA, 1.

LE SALLE, M. & BILLEN, D. (1964). Inhibition of DNA synthesis in murine bone-marrow cells by AET and cysteamine. *Ann. New York Acad. Sci.*, **114**, 622.

MEYN, R.E. & JENKINS, W.T. (1983). Variation in normal and tumor tissue sensitivity of mice to ionizing radiation-induced DNA strand breaks in vivo. *Cancer Res.*, **43**, 5668.

MILAS, L., HUNTER, N., STEPHENS, C.L. & PETERS, L.J. (1984). Inhibition of radiation carcinogenesis by S-2-(3-aminopropylamino)-ethylphorothioic acid. *Cancer Res.*, **44**, 5567.

MURRAY, D., JENKINS, W.T. & MEYN, R.E. (1984). The efficiency of DNA strand-break repair in two fibrosarcoma tumors and in normal tissues of mice irradiated *in vivo* with X rays. *Radiat. Res.*, **100**, 171.

NAGY, B., DALE, P.J. & GRDINA, D.J. (1986). Protection against cis-diamminedichloroplatinum cytotoxicity and mutagenicity in V79 cells by 2-[(aminopropyl)amino] ethanethiol. *Cancer Res.*, **46**, 1132.

NAGY, B. & GRDINA, D.J. (1986). Protective effects of 2-[(aminopropyl)amino] ethanethiol against bleomycin and nitrogen mustard-induced mutagenicity in V79 cells. *Int. J. Radiat. Oncol. Biol. Phys.*, **12**, 1475.

PERAINO, C., STAFFELDT, E.F., CARNES, B.A., LUDEMAN, V.A., BLOMQUIST, J.A. & VESSELINOVITCH, S.D. (1984). Characterization of histochemically detectable altered hepatocyte foci and their relationship to hepatic tumorigenesis in rats treated once with diethylnitrosamine within one day after birth. *Cancer Res.*, **44**, 3340.

PHILLIPS, T.L. (1980). Rationale for initial clinical trials and future development of radioprotectors. *Cancer Clin. Trials*, **3**, 165.

PURDIE, J.W. (1979). A comparative study of the radioprotective effects of cysteamine, WR-2721, and WR-1065 in cultured human cells. *Radiat. Res.*, **77**, 303.

RUSSEL, W.C., NEWMAN, C. & WILLIAMSON, D.H. (1975). A simple cytochemical technique for demonstration of DNA in cells infected with mycoplasma and viruses. *Nature*, **253**, 461.

SAWADA, S. & OKADA, S. (1970). Cysteamine, cystamine, and single-strand breaks of DNA in cultured mammalian cells. *Radiat. Res.*, **44**, 116.

SLAGA, T.J., KLEIN-SZANTO, A.J.P., TRIPLETT, L.L., YOTTI, L.P. & TROSKO, J.E. (1981). Skin tumor-promoting activity of benzoyl peroxide, a widely used free radical-generating compound. *Science* (Wash.), **213**, 1023.

SUZUKI, F., HAN, A., LANKAS, G.R., UTSUMI, H. & ELKIND, M.M. (1981). Spectral dependencies of killing, mutation, and transformation in mammalian cells and their relevance to hazards caused by solar ultraviolet radiation. *Cancer Res.*, **41**, 4916.

THACKER, J. (1986). The nature of mutants induced by ionising radiation in cultured hamster cells. III. Molecular characterization of HPRT-deficient mutants induced by $\gamma$-rays or $\alpha$-particles showing that the majority have deletions of all or part of the HPRT gene. *Mutation Res.*, **160**, 267.

YUHAS, J.M., SPELLMAN, J.M. & CULO, F. (1980). The role of WR2721 in radiotherapy and/or chemotherapy. *Cancer Clin. Trials*, **3**, 221.